# The lived experience of long-term follow-up clinical care for haematopoietic stem cell recipients in England: a qualitative exploration

**DOI:** 10.1007/s11764-023-01399-w

**Published:** 2023-05-16

**Authors:** Blossom Bell, Katherine Swainston

**Affiliations:** Newcastle University, Newcastle Upon Tyne, UK

**Keywords:** Stem cell transplant, Patients’ experiences, Long-term monitoring, Late effects

## Abstract

**Purpose:**

Despite a haematopoietic stem cell transplant (HSCT) being a potentially curative treatment option for malignant and non-malignant disorders, patients may develop complex physical and psychological post-transplant complications. Consequently, transplant centres remain responsible for patients’ life-long monitoring and screening practices. We sought to describe how HSCT survivors experience long-term follow-up (LTFU) monitoring clinics in England.

**Method:**

A qualitative approach was adopted with data collected from written accounts. Seventeen transplant recipients were recruited from across England, and the data was analysed using thematic analysis.

**Results:**

Data analysis elicited four themes: Transfer to LTFU care: ‘will there be a change in my care, or will appointments just become less frequent?’; Care Coordination: ‘it is good to know I am still in the system’; Relationship continuity: ‘a good knowledge of me, my health and what is important to me’; and Late-effects Screening: ‘there was not much information about what to expect or be aware of’.

**Conclusions:**

HSCT survivors in England experience uncertainty and lack of information regarding the transfer from acute to long-term care and clinic screening practices. However, patients gain reassurance from remaining on a healthcare pathway and maintaining relationships with healthcare professionals.

**Implications for cancer survivors:**

HSCT recipients entering LTFU monitoring clinics are a growing population of cancer survivors. Understanding and acknowledging this cohort of patients’ needs may inform the development of tailored support to help patients navigate the complicated healthcare pathway.

## Background

A haematopoietic stem cell transplant (HSCT) is a potentially curative treatment option for malignant and non-malignant haematological disorders, such as leukaemia, lymphoma, and hemoglobinopathies [1]. The standard procedure involves eradicating a patient’s bone marrow cells (myeloablation) using high-dose chemotherapy, sometimes combined with radiotherapy (conditioning), and then intravenously reintroducing healthy haematopoietic stem cells to restore normal marrow cellularity. Replacement haematopoietic stem cells can either be obtained from the peripheral blood or bone marrow of the patient (autologous transplant) or a donor (allogeneic transplant). Donor haematopoietic stem cells can also be obtained from umbilical cord blood [2].

Despite being a high-cost and specialised procedure, the prevalence of HSCT procedures performed worldwide continues to rise [3]. In the United Kingdom (UK) alone, 3566 HSCTs were performed in 2020, a 63% increase compared to the number undertaken in 2000 [4]. This increase is attributed to advancements in immunogenetics and immunobiology, reduced intensity conditioning regimens, disease identification and clinical risk, and patient management [5, 6]. Furthermore, these attributes have contributed to decreased transplant-related mortality, especially in the early (day + 100) and intermediate (+ 1 year) post-transplant phase. However, for allogeneic recipients, mortality remains high decades post-HSCT, with life expectancy remaining below that of the general population [7, 8]. Survivors are at risk of developing late complications, such as secondary cancers, late infections, graft-versus-host disease (GvHD), and psychosocial issues, which can manifest months to years after HSCT [9]. The detrimental effect of post-transplant complications (i.e. late-effects) has consequently shifted focus to the health management and preventative practices of HSCT survivors by transplant centres and other healthcare professionals [10].

In England, there are currently twenty-eight designated centres providing HSCT, which the National Health Service (NHS) England (i.e. the NHS lead in England) states must have accreditation in accordance with the Foundation for the Accreditation of Cellular Therapy and the Joint Accreditation Committee International Society for Cellular Therapy and European Society for Blood and Marrow Transplant Research standards [11]. Since 2015, the accreditation standards have specified that transplant centres must have policies or standard operating procedures for long-term follow-up (LTFU) care and monitor recipients for post-transplant late effects. As a minimum, either directly or coordinated with primary care providers, transplant centres are required to monitor recipients endocrine, reproductive and respiratory functions, cardiovascular risk factors, and renal impairment, as well as monitor for signs of osteoporosis and secondary cancers [12]. To assist with the management of LTFU patients’ needs and optimise efficiency as recipients transition from the acute post-transplant phase to LTFU care, the standards recommend the establishment of dedicated clinics and endorse specific sources of reference when establishing clinical guidelines, including the international guidance on recommended screening and preventative practices for long-term HSCT survivors and the integration of these guidelines into clinical practice [13, 14].

Despite the acknowledgement that the efficiency of the transition from acute post-transplant care to LTFU care is essential to the quality of care received and that a multi-disciplinary approach to care should be employed [15], the provisions for LTFU care for HSCT patients are known to be variable [16, 17]. In the UK, there is a dearth of research examining the implementation of LTFU provisions, with only one study being identified. Twenty-five allogeneic transplant centres in the UK, of which 21 were in England, responded to a 2019 web-based survey examining service organisation, access to specialist services, including screening and vaccination, and team and patient engagement of LTFU provisions [18]. Twenty-one (84%) transplant centres had developed a dedicated LTFU clinic, whilst one centre referred recipients to general haematology outpatients for monitoring, another had no formal LTFU clinic, and two centres failed to answer the question. A hybrid approach, between primary or secondary care providers and transplant centres, to LTFU care was adopted by 44% of centres. Monitoring is evidently taking place; however, deficiencies exist, including limited access to services, biased screening activities limited to within transplant physicians’ control, and lack of psychological support. Insufficient financial resources, different healthcare structures, and transplant centres specific interests in LTFU were reported as possible reasons for the deficiencies. Further research correlating LTFU services with patient-reported outcomes was recommended as one potential solution to addressing current deficiencies in services.

Literature observing the LTFU monitoring from recipients’ perspectives post-2015 is limited, with only three qualitative studies identified [19–21]. All three studies found that recipients established close relationships with clinical personnel, which was seen as a crucial element to receiving personal and attentive care, as well as maintaining health. Furthermore, recipients identified several logistical problems with attending monitoring clinic appointments, such as delays, length, frequency, and financial consequences. Studies found that recipients reported a fear of recurrence and uncertainty of the future, feelings which were associated with monitoring practice. However, variation in country-specific and hospital healthcare structure does not allow for the findings to be generalised to HSCT recipients based in England. Furthermore, two studies recruited recipients from dedicated GvHD clinics; therefore, patients may be under active care resulting in more attendance requirements and heightened awareness.

At present, it remains unknown how HSCT survivors experience LTFU care within the NHS England care structure. This poses a barrier as it cannot be determined if transplant centres are adequately and effectively implementing long-term monitoring requirements from a recipient’s perspective, especially considering the known deficiencies reported by transplant centres. Given the paucity of knowledge and lack of UK-based research since the 2015 accreditation change, this study aims to explore the experiences of LTFU care services from the perspective of HSCT recipients treated in centres based in England.

## Methods

### Design

A qualitative design underpinned by a phenomenological approach was adopted to understand the lived experiences of post-transplant LTFU clinical care for HSCT recipients. The philosophy of phenomenology reinforced the methodological principles, to transform through reflection on the meaning, quality, and texture of participants’ experiences [22]. This involved analysing participants’ experiences from their perspective through their thoughts and feelings and identifying recurring themes across the data set. Open-ended written accounts of experiences were obtained online, a method chosen due to its ability to capture a ‘wide-angle’ spread and diversity of experiences within an unexplored population [23].

### Participants

Seventeen recipients (7 males, 10 females) of HSCT were recruited via purposive sampling from across England. Sample size guidelines suggest a range of between 15 and 50 qualitative written accounts for participant-generated textual data of life experiences. It was deemed on receipt of 17 written accounts that data saturation had been reached as no further themes were generated from the data [24]. The inclusion criteria were that participants were at least 18 years of age at the time of data collection and at least 1-year post-transplant to ensure the long-term monitoring phase of treatment had been reached. Participants’ age at transplant ranged from 3 to 65 years (mean age 44.64 years), and the average time since transplant was 8.24 years (range 1.16–33.58 years) (see Table 1). Thirteen participants received LTFU care from a designated LTFU clinic, two attended general haematology outpatient’s department for LTFU, whilst a further two received a hybrid approach where LTFU care is provided by both a designated LTFU clinic and primary care providers. Of the seventeen participants, fifteen received LTFU care at the same hospital as their transplant, eight of which were reported as being their local hospital. Two participants receive LTFU at a different hospital from where they had their transplant; for one, this was their local hospital. In total eight participants did not receive LTFU care at their local hospital and must travel to attend, a distance which ranged from 5.5 to 70 miles one way (mean distance of 34.4 miles). The interval between LTFU appointments at the time of data collection ranged from once every 4 weeks to once every 5 years (mean time once every 9 months).

**Table 1:** Participant characteristics

Participant number	Gender	Age at transplant	Transplant type	Time since transplant (years)	LTFU monitoring frequency	LTFU care model	LTFU at same hospital as transplant
1	Male	55	Allo PBS	8.3	Yearly	Clinic	Yes
2	Female	60	Allo PBS	2.8	3 monthly	Hybrid	Yes
3	Female	50	Allo PBS	2.7	6 monthly	Clinic	Yes
4	Female	28	Allo BMT	27.1	5 Yearly	Clinic	Yes
5	Male	43	Allo BMT	6.4	8 weekly	Clinic	Yes
6	Male	63	Allo PBS	4.4	6 monthly	Clinic	Yes
7	Female	61	Allo BMT	3.7	3 monthly	Haem OP	Yes
8	Male	49	Allo BMT	4.8	Yearly	Clinic	Yes
9	Male	65	Hap SCT	1.2	8 weekly	Clinic	Yes
10	Female	42	Allo BMT	10.1	3 monthly	Clinic	Yes
11	Female	3	Auto BMT	33.6	Yearly	Clinic	No
12	Male	50	Hap SCT*	10.9	2 yearly	Clinic	Yes
13	Female	34	Allo PBS	1.6	2 monthly	Hybrid	Yes
14	Female	54	Allo BMT	1.1	4 weekly	Clinic	Yes
15	Female	31	Allo PBS	2.7	3 monthly	Clinic	Yes
16	Male	25	Auto BMT*	10.8	Yearly	Clinic	Yes
17	Female	46	Allo PBS	7.8	Yearly	Haem OP	No

*multiple transplants with the last type received indicated; *Allo PBS*, allogeneic peripheral blood stem cell transplant; *Allo BMT*, allogeneic bone marrow transplant; *Hap SCT*, haploidentical stem cell transplant; *Auto BMT*, autologous bone marrow transplant; *Clinic*, designated LTFU clinic; *Hybrid*, coordinated care between LTFU clinic and primary care providers; *Haem OP*, haematology outpatients

### Materials

Materials comprised a participant information sheet, consent form, instruction sheet, and debrief. The written account document comprised one open-ended question: ‘Please tell me about your experiences of attending designated long-term late-effects monitoring appointments following stem cell transplantation’. Further instruction was provided as suggestions for areas of reflection: ‘You may want to consider areas such as: how and when you entered long-term monitoring; details of appointment arrangements; appointment objectives; feelings and thoughts prior to and after each appointment; services expectations; how you feel about the care you receive; advantages and disadvantages to health; any issues or concerns; benefits of attending; future expectations etc.’. Participants could amend/add to their accounts as many times as required prior to final submission. Demographic data, collected for the purposes of providing sample information only, included gender, type of HSCT, month and year of transplant, age at transplant, if LTFU appointments occurred at their local hospital, and if not, then how far they have to travel, whether LTFU occurred at the same hospital as their transplant, and how often then attend LTFU appointment.

### Procedure

Following ethical approval from Teesside University, the study materials were distributed via Jisc Survey Online (JSO). Two social media private group pages, one for survivors of HSCT and another for survivors of a condition often treated with HSCT, gave permission to post a recruitment advert for the study. In addition, a UK-based charity promoting stem cell donation advertised the study via their social media page. All adverts provided a link to JSO. On accessing the link, participants were initially provided with the participant information sheet and then the consent form. Once consent was given, participants were asked the demographic data questions followed by the open-ended question. Once written accounts had been submitted, all participants were provided with a debrief sheet which summarised the study and provided contact details for further support.

### Data analysis

The data were analysed using thematic analysis, chosen due to its flexibility and suitability to evaluate a wide range of data items, including textual data [25, 26]. The inductive approach allowed for the identification of themes and patterns and analysed. To ensure the themes adequately represented the data set, the researcher engaged in reviewing and defining the themes, ensuring the themes represent the coded data, the central theme, and the whole data set. It should be acknowledged that the lead researcher (BB) has direct experience with HSCT post-transplant and late-effects monitoring. A reflective journal was maintained throughout, and supervision was provided throughout the research process by KS. A second analysis of the data was undertaken by KS, and all members of the research team were involved in refining and naming themes.

## Findings

Four themes were drawn from the data analysis: transfer to LTFU care: ‘will there be a change in my care, or will appointments just become less frequent?’; Care Coordination: ‘it is good to know I am still in the system’; Relationship continuity: ‘a good knowledge of me, my health and what is important to me’; and Late-effects Screening: ‘there was not much information about what to expect or be aware of’.

### Theme 1: Transfer to LTFU care: ‘will there be a change in my care, or will appointments just become less frequent?’

Participants’ accounts described two different appointment practices: post-transplant and LTFU monitoring. Central to experiences was uncertainty surrounding the official transfer between follow-up and monitoring. Participants reported being unsure if transfer between monitoring practices would be acknowledged by receiving a change in care or implied through decreased frequency between appointments.

[I] do not feel that I’ve officially entered ‘long term’ follow-up yet. I am not sure if this will be officially marked by a change in my care or whether my appointments will just become less frequent. (P13)

LTFU, for some participants, was stated or perceived to have commenced when they experienced a change in treatment needs.

I had both transplants at [stated hospital] but unfortunately relapsed from both quite quickly, it was only a combination of [low-dose irradiation] [and] monoclonal antibody therapy [for chronic graft-versus-host disease] that I am in remission now. I only started long-term effects clinics about five years ago after the [chronic graft-versus-host disease] had finally settled down. (P16)

However, active treatment for post-transplant complications prevented clinical late-effects discussions from occurring, leading participants to question if any long-term monitoring had been received.

I’m not sure that my long-term / late effect monitoring has happened… I had an early relapse and then another several years later… we didn’t talk about late effects as there was still too much going on. (P10)

For others, LTFU monitoring appeared to have commenced when the clinical responsibility was transferred to another healthcare provider and/or when appointment frequency gradually decreased to annually.

Not sure when long-term monitoring started… I was transferred by transplant hospital to a local hospital 4 years after the transplant and this obviously marked long-term follow-up as appts went to yearly. (P17)

The uncertainty participants described surrounding the official or perceived commencement of LTFU monitoring was mitigated by the acknowledgement of remaining within a healthcare pathway, regardless of the care mode employed.

### Theme 2: Care Coordination: ‘it is good to know I am still in the system’

Participants described three different modes of care coordination, and whilst the experiences varied, generally, participants expressed gratitude for remaining within a healthcare pathway regardless of the mode of delivery and experienced less perceived anxiety.

It is good to know I am ‘still in the system’ rather than being signed off as I think this would make me more anxious. (P3)

Most participants had LTFU care activities organised and executed by a specialist team within a designated transplant centre. Planned care activities included regular blood testing, medical referrals, and prescription distribution. Participants appreciated the multi-disciplinary approach for its efficiency and effectiveness.

The transplant nurses and pharmacy assistants are always available in the clinic, and this makes the whole visit process work very smoothly as they can book anything and order drugs all on the same day. (P14)

Furthermore, the competency of service provided by the LTFU clinics removed general practitioner (GP) contact and involvement in post-transplant care.

I have had little contact with my local GPs since most issues were dealt with by the haematology department, including long-term prescriptions. (P1)

However, the appointment structure influenced the participants’ experiences. Participants reported tiresome waits between appointments, and whilst waiting was an accepted part of the process, it was exacerbated by travel requirements and work commitments, especially for participants attending clinic(s) not local to their residential location.

Sometimes it can take a long time in the clinic – i.e., to have blood done and then have to wait to see the consultant, so that can be tiring, but it’s a necessary evil. (P14)

I find them very organised, but it is time-consuming - only on a Wednesday morning and generally takes the best part of the day [when including] travel. (P3)

For others, care activities were provided through a hybrid approach: transplant centres maintained the clinical responsibility by holding consultations remotely but relied on primary healthcare providers to carry out tests, especially in relation to blood tests, with participants only having to attend transplant centre for specialist tests. Participants found the approach convenient and flexible to their needs.

[LTFU clinic] has liaised well with a more local hospital allowing me to have blood tests locally and have my consultations by phone when this is more convenient and I am able to request phone or face-to-face to suit me. (P13)

The approach became more frequent during the pandemic, which participants reported preferring as it removed stress associated with travelling to the hospital and reduced waiting times.

Although the distance isn’t horrendous, the journey is, and I find it stressful. But because of covid, most appointments are now video calls, which I prefer... All blood tests are now done at my doctor’s surgery prior to video calls. (P2)

However, for some, the lack of physical attendance impacts health anxiety symptoms, and physically being seen was described as alleviating these symptoms.

I have a lot of health anxiety, so attending these appointments is helpful in alleviating worries and I feel better having physically seen someone and been checked. (P15)

For another participant, the responsibility for LTFU healthcare needs was transferred to their primary care providers in its entirety. This transfer resulted in a loss of a specialist and a multi-disciplinary approach to care coordination, which impacted the participant’s confidence regarding their future health and heightened their desire for specialist support via telephone or email, a service available to other participants. Having immediate and direct contact with clinical personnel was reported to be an essential supportive service, especially if enquiries were of a concerning or urgent nature and associated with receiving efficient and effective care.

My confidence going forward in relation to my health is non-existent right now, which impacts my mental well-being…if there were support, telephone or email, with a post-transplant specialist, I would feel so much better as I would have that reassuring support when needed, but instead, I often end up quite unnecessarily unwell for much longer. (P17)

I have access to a [clinical nurse specialist] by phone and email, which is absolutely essential to my feeling of being looked after and knowing that I can contact somebody with any concerns. (P13)

Remaining within the post-transplant care pathway, regardless of the mode of delivery, was essential to participants’ experiences and obtaining supporting needs: a pathway strengthened by a continuous relationship with healthcare providers.

### Theme 3: Relationship continuity: ‘a good knowledge of me, my health and what is important to me’

Some participants experienced a continuous relationship with LTFU healthcare professionals that have developed over time and remained consistent. For some, this included a relationship with the same consultant that oversaw the transplant that had continued into LTFU care. This continuity was reported to be an essential component of receiving efficient and attentive care that was holistic in nature. Receiving this care combined with expert knowledge was reported to mitigate any inconvenience caused by attending.

I usually see my main consultant, who has seen me throughout my transplant process, and this is helpful as he has a good knowledge of me, my health and what is important to me. (P15)

I have developed a good relationship and rapport with the doctors I see in the clinic. They always impress me with their sensitive and intelligent approach to their patients…There is often a wait to be seen but that is no problem for such high-quality advice. (P1)

For other participants, a prolonged continuous relationship was established with clinical nurse specialists, rather than consultants, and contributed to participants receiving conscientious and attentive care whilst in appointment attendance.

I get seen by a specialist nurse whom I have known for a long time, they are very thorough, and I feel well looked after when I am there. (P16)

However, frequent and continuous relationships with clinical personnel prevented participants from seeking healthcare assistance from primary healthcare providers, specifically GPs. This reluctance is linked to a perceived notion that GPs have insufficient expertise to manage long-term monitoring requirements due to the complicated nature of late effects.

As I still speak to my team fairly regularly, I’ve found myself raising most medical issues with them and have not considered going to my GP for anything else… a stem cell transplant has so many potential effects that a GP might not be able to rule out transplant-related issues… So, the transplant team becomes like a surrogate GP. (P13)

Furthermore, liaising with GPs was stated as being futile as they would inadvertently liaise with the TC.

I’m very happy with the support I get from my [the clinical] team – They’re friendly, helpful and responsive, and I would much rather talk to them than a GP as GPs don’t have the experience and always end up calling the [hospital] anyway. (P7)

However, for one participant, the transfer of long-term monitoring responsibilities to their local and non-transplant hospital resulted in the loss of the continuous relationship previously experienced whilst under the care of the transplant team and expected to be adopted at the new centre. However, this absent relationship resulted in a loss of trust and feelings of abandonment, as well as contributed to the insufficient implementation of the long-term monitoring guidelines.

At my last [appointment] in the new hospital and the first [appointment] seeing a [new] consultant… he dismissed everything I said or asked about and I felt like he had belittled my experiences… The care that I received in my transplant hospital was on the whole good in the sense I was listened to, so I thought this would continue but feel that late effects clinics are just lip service or a tick box exercise. (P17)

### Theme 4: Late-effects Screening: ‘there was not much information about what to expect or be aware of’

Patients reported a lack of preparation from hospitals regarding the time frame of recovery following the transplant.

I don’t think the hospital really prepares you for how long the whole transplant recovery process can really take… but overall I think the care has been and still is very good. (P14)

Furthermore, participants reported uncertainty about the amount of information they received regarding the possible late effects or what participants could expect from attending monitoring appointments.

I don't think I was told about some of the possible late effects, and I don’t think there was much information about what to expect or be aware of. (P10)

For some, the knowledge of late effects developed over time either due to the personal development of late-effect complications or because of the evolving list of recognised medical late effects.

Having had my transplant [27 years] ago, I can say that over recent years more and more late effects have been noticed… When I first had my transplant, there weren’t many recognised late effects apart from infertility and long-term graft-versus-host disease. (P4)

For others, late-effects knowledge developed from discussions with LTFU clinic personnel, which focused on specific age determined screening tests. However, participants report lack of confidence in clinics remembering to action the tests at the appropriate time age interval, resulting in participants being proactive in their care needs.

I have not yet reached many of the ‘milestones’ at which specific late effects need to be monitored for, however these are mentioned from time to time and I try to write them down as I don’t always feel confident that this will be remembered at the right time by my medical team. (P13)

In addition, participants report still waiting for screening tests discussed in clinic to occur or expressed difficulty in obtaining. This impacted participants’ concerns in receiving potential future screening tests, especially if such tests are not considered life limiting.

Some of the follow up discussed in the late effect clinic hasn’t happened yet. (P10)

I’ve had a difficult experience trying to get referred for a fertility assessment, which is distressing in itself but also makes me worry about other things I might need in the future, which aren’t a matter of life and death but do affect quality of life greatly. (P13)

The need to take ownership of their own care needs for some participants was stated to occur due to the reported knowledge of the inconsistent approach to monitoring practices across centres. This included taking control of obtaining medical referrals to specialist clinics, underpinned by concerns regarding the impact of treatment on health.

I have been proactive in getting myself referred to the [hospital] to a specific cardio-oncology service… I know now that people who are on the drug I went on are now routinely offered more monitoring at some hospitals, e.g. annual heart check, whereas I didn’t have that kind of thing, so things maybe lay undetected for some time. (P10)

Furthermore, the unknown impact of treatment on future health was stated as contributing to pre-appointment anxiety for screening tests, intensified by waiting times.

I’m on the waiting list for a bone scan and I’m a bit concerned about this because I don’t know how good my bones are because of early menopause and cardiac complications. (P10)

Participants also reported experiencing pre-appointment anxiety prior to attending LTFU appointments due to concerns regarding post-transplant complications, specifically relapse. The anxiety experienced is enhanced by the pending examination of screening test and the scrutinisation of results.

I still feel quite shellshocked several years after diagnosis and anxious in the lead-up to appointments [regarding] relapse. This anxiety is increased by… wondering what consultants and I should be looking out for in the blood results. (P3)

The limited knowledge of late-effects and participants’ desire to take ownership and be proactive in post-transplant screening activities were described as warranting more support. Having written documentation identifying and detailing future monitoring and screening requirements was highlighted as a potential beneficial need.

## Discussion

The aim of this study was to explore haematopoietic stem cell transplant recipients’ experiences of long-term follow-up monitoring across England. The themes generated from the patients’ written accounts of their experiences were the uncertainty regarding the transfer to LTFU care, reassurance gained from remaining on a healthcare pathway, efficient and effective continuous relationships with healthcare professionals, and the lack of information and expectation surrounding late-effect screening practices. The findings are consistent with the results of previous studies that close personal relationships with clinics were essential to receiving personal and attentive care to maintain health and that for some patients fear of recurrence and uncertainty was associated with monitoring practices [19–21]. However, the patients’ accounts depict the complexities, uncertainty, and psychological impact on HSCT recipients as they navigate the LTFU healthcare pathway to ensure continuity of care.

An important finding of this study is the lack of primary care providers, specifically GPs, involvement in LTFU care. Participants reported either not seeking GP involvement due to clinics meeting their health needs or a reluctance based on their perception that primary care providers do not possess the necessary experience and knowledge to monitor LTFU care efficiently. Regardless of the underlying cause, the lack of involvement of primary care providers in LTFU care may become problematic for transplant centres in the future, as the continued rise in the number of transplants performed annually, combined with transplant centres’ responsibility to coordinate LTFU care and the more frequent use of a hybrid approach, may increase pressures on services. It is, therefore, essential that primary care providers working within the hybrid approach have the necessary knowledge and expertise of the late effects that can arise after HSCT, which is essential in delivering efficient survivorship care [15] and merits further research. This is especially  important considering the NHS Long Term Plan to incorporate a Personalised Stratified Follow-Up pathway within all cancer services, which aims to provide support closer to patients’ residential locations, implying more ownership on primary care providers to provide LTFU care [27]. First, future research may consider exploring the current level of knowledge held by primary care providers and identifying potential educational and training needs, if required. As for recipients’ perception of primary care providers, the present study design of written accounts did not allow for exploration within the data collective phase; therefore, further research in this area would be beneficial.

The findings of this study suggest that primary consultants are not discussing the transition process with HSCT survivors, given the ambiguity surrounding the official commencement of LTFU from acute post-transplant care for participants, as recommended in the guidance literature [13, 14]. This results in a loss of continuity of care for some patients; a direct contradiction to the accreditation standard’s aims to prevent losing patients at follow-up [12]. To facilitate the transition from acute to LTFU care and to ensure continuity of care for patients, Hashmi et al. (2015) and Majhail et al. (2012) recommend providing patients with survivorship care plans. Such plans, like the treatment summary and survivorship care plan created by the American Society of Clinical Oncology [28], should define the roles and responsibilities of preventative care providers to optimise LTFU efficiency and serve as a reminder for consultants and patients of the appropriate surveillance practices tailored to individual risk factors. However, since patients in this study highlighted a need for written documentation identifying and detailing future monitoring and screening requirements suggests that such an instrument remains unavailable and worthy of further investigation. Future research may consider exploring the availability and content of survivorship care plans among a wider and demographic diverse sample of participants. Although this sample represented HSCT recipients, recruiting via online support groups limited the reach of participants to only those participating in social media, which may have resulted in a less demographically diverse and biased sample as recruiting participants online inadvertently excludes HSCT patients not actively engaged in online social media platforms [23, 29].

Survivorship care plans could also support the psychological impact some recipients in this study report experiencing from attending LTFU appointments. Attending appointments, receiving blood count results, and waiting for clinical tests have all been stated as a source of anxiety for some recipients, and it is largely the uncertainty surrounding each entity that gives rise to this anxiety. The lack of psychological support offered by clinics could be why recipients are experiencing this impact [18]. However, as patients report less anxiety when they have a low level of informational needs, patients should be encouraged to participate in their own long-term care since attitudes towards their illness, treatment, and health behaviours are an important factor [30]. There is little reference within the guidelines to the health behaviours of recipients and how health behaviour can assist with the monitoring services, especially regarding the promotion and maintenance of health. Further research in the area and the potential application of health promotion within survivorship care plans is warranted. However, the implications of highlighting long-term health after HSCT with a hypervigilant cohort of patients must be considered, and adequate support must be implemented to ensure no detriment to psychological well-being.

In addition to those previously stated, there are several limitations to this study. Firstly, whilst all care models were represented in the sample and data saturation was met at 17 participants, the study would have benefited from a larger sample size to convincingly demonstrate patterns across the data set, specifically the data provided by the haematology outpatients and hybrid care model group [24]. In addition, not all participants provided data regarding their demographic location, preventing the identification of any potential regional difference within the sample. Secondly, whilst utilising open-ended written accounts allowed recipients to express matters important to them whilst providing the researchers access to their language and terminology, reducing the potential of being lost in transcription [23], incorporating prompts may have limited the freedom of expression. Recipients may have felt required to answer the question rather than document their experiences as prioritised in their thoughts in full. Thirdly, the post-transplant care pathway is not uniform for all patients; the complexity of late effects means that there is potential that some recipients were still under active care when completing the written account. Finally, some participants received transplants prior to the 2015 accreditation change; therefore, there is no responsibility on transplant centres to monitor their LTFU care; consequently, some accounts may not reflect recipients’ experiences transplanted post 1 June 2015.

In conclusion, based on the written accounts of HSCT patients included in this study, uncertainty regarding the transfer to LTFU care, reassurance gained from remaining on a healthcare pathway, efficient and effective continuous relationships with healthcare professionals, and the lack of information and expectation surrounding late-effect screening practices summarises their experiences in long-term monitoring clinics in England. Opportunities exist to extend future research to understand the impact of each finding further and should focus on three key areas; (1) exploring primary care providers existing knowledge of late-effects and patients’ experiences of using primary care providers for LTFU care, (2) the availability and content of survivorship care plans for HSCT recipients, and (3) exploration of recipients’ health behaviours post-transplant and relationship with monitoring services, to gain a greater understanding of HSCT patient supports needs.

## Data Availability

All data generated or analysed during this study are included in this published article.
